# p16INK4a Deletion Alleviated Obesity‐Associated Kidney Fibrosis by Regulating Metabolic Reprogramming and the Inflammasome Pathway

**DOI:** 10.1111/jcmm.70444

**Published:** 2025-03-13

**Authors:** Qian Liu, Fen Wang, Yuan Du, Yankui Liu, Zhixuan Zhang, Xiaodong Zhang, Jianwei Li, Guangyi Huang, Fengqi Liu, Biahong Li, Wang Xiao, Chenyan Sui, Neng Bao, Ruijuan Zhuang, Changzheng Gao, Xiaoyan Wang, Xin Gu

**Affiliations:** ^1^ Department of Cardiology The Affiliated Hospital of Jiangnan University Wuxi Jiangsu China; ^2^ Department of Nephrology The Affiliated Hospital of Jiangnan University Wuxi Jiangsu China; ^3^ Department of Pathology The Affiliated Hospital of Jiangnan University Wuxi Jiangsu China; ^4^ Department of Neurology The Affiliated Hospital of Jiangnan University Wuxi Jiangsu China

**Keywords:** cell senescence, kidney fibrosis, metabolic remodelling, p16INK4a

## Abstract

Recent research has revealed a close association between obesity and various metabolic disorders, including renal metabolic diseases, but the mechanism is still unknown. This study explored the role of p16INK4a in obesity‐related kidney fibrosis and evaluated its potential as a therapeutic target. Using wild‐type (WT) mice and p16 KO mice, we fed both groups a high‐fat diet (HFD) for 6 months. Our results showed that an HFD led to significant weight gain and increased p16INK4a expression in WT mouse kidneys. Notably, p16 KO mice presented reduced fibrosis, as indicated by decreased levels of profibrotic proteins (α‐SMA and collagen I) and improved histological outcomes, including reduced fibrosis in the glomeruli and renal tubules. P16 KO also suppressed the levels of several proinflammatory biomarkers (MMP1, MMP3, IL‐1β, TNF‐α and IL‐6) and inhibited the NLRP3 inflammasome pathway. The administration of ABT263 further validated these findings by decreasing fibrosis and inflammation in HFD‐fed mice, suggesting that p16INK4a contributes to both fibrotic and inflammatory processes. Metabolomic analyses revealed that p16 knockout influenced various metabolic pathways, including linoleic acid and pyrimidine metabolism, in HFD‐induced kidneys. Additionally, p16INK4a over‐expression was observed in the kidneys of chronic kidney disease patients with long‐term hyperlipidaemia. These results highlight the critical role of p16INK4a in obesity‐induced kidney damage and suggest that targeting p16INK4a may be a promising approach for treating obesity‐related kidney fibrosis and inflammation.

## 1 Introduction

The increasing prevalence of obesity contributes significantly to a range of diseases, including cardiovascular and metabolic disorders, which result in millions of fatalities annually [1, 2]. Recent research has revealed a close association between obesity and various metabolic disorders, including renal metabolic diseases [3]. Paredes et al. [4] reported that a high‐fat diet (HFD) can induce both obesity and kidney dysfunction in a rat model. Previous investigations have linked renal insufficiency induced by high‐fat diets to mechanisms such as renal fat accumulation and heightened inflammatory responses [5, 6]. However, the precise mechanisms by which obesity‐related renal dysfunction is mediated by high‐fat diets remain inadequately understood and warrant further exploration.

The accumulation of senescent cells within the microenvironment is known to precipitate chronic low‐grade inflammation and excessive accumulation of pro‐inflammatory biomarkers, contributing to inflammation across multiple tissues [7, 8]. Senescent cells secrete senescence‐associated secretory phenotype (SASP) factors that impact neighbouring cells and tissues. Emerging evidence suggests that cellular ageing plays a critical role in the pathophysiological mechanisms underlying kidney injury [9, 10]. Prior research has shown the accumulation of senescent cells in the kidneys of premature knockout mice as well as their involvement in kidney fibrosis and the epithelial–mesenchymal transition (EMT) process within renal tubules [11]. Additionally, recent studies have demonstrated that ABT263, a small molecule that targets senescent tubular cells, can effectively promote kidney repair and mitigate cisplatin‐induced chronic kidney disease (CKD) [12].

p16ink4a (p16) functions as an inhibitor of the cyclin‐dependent kinase (CDK) complex CDK4/6, resulting in decreased phosphorylation of the retinoblastoma protein (Rb). This inhibitory action halts the progression of the cell cycle from the G1 phase to the S phase, thus arresting cell proliferation and inducing cellular senescence. Elevated expression of p16 is observed in renal tissues exhibiting proteinuria, fibrosis or interstitial inflammation [13]. Our previous research demonstrated that p16 knockout (KO) reduces the number of senescent cells and diminishes the expression of various cytokines in high‐fat diet‐induced lung fibrosis [8]. Moreover, p16 knockout has been shown to attenuate kidney dysfunction and fibrosis in Bmi1 knockout mice, likely through its inhibitory effects on oxidative stress and DNA damage response pathways [11, 14]. Despite these findings, the role of p16 in obesity‐associated renal diseases remains inadequately defined.

This study aimed to investigate whether p16 exacerbates the severity of obesity‐related renal dysfunction and to elucidate the protective mechanisms by which p16 gene deletion influences renal cell senescence and kidney injury. We used 14‐month‐old p16 KO mice and wild‐type (WT) mice and subjected them to a high‐fat diet for 6 months. Our findings indicate that p16 gene knockout alleviates pathological changes associated with obesity‐related kidney fibrosis. P16 KO inhibited the activation of the inflammasome pathway and induced metabolic reprogramming in the kidneys. Additionally, the targeted elimination of p16‐positive senescent cells using ABT263 ameliorates obesity‐related renal fibrosis. These results underscore the critical role of p16 in obesity‐related nephropathy and suggest that p16 may serve as a potential target for therapeutic interventions aimed at improving kidney disease outcomes in the future.

## 2 Materials and Methods

### 2.1 Study Animals and Patients

Exon 1α of p16 was knocked out. p16 heterozygote male and female mice of the FVB N2 background were mated to generate p16 KO mice and WT littermates genotyped. We purchased 8‐week‐old male ApoE knockout (ApoE^−/−^) mice from the Model Animal Research Center, Nanjing University and then mated them with p16^−/−^ mice to generate ApoE^−/−^ p16^−/−^ animals. In this experiment, we used 14‐month‐old male ApoE^−/−^ and ApoE^−/−^p16^−/−^ mice [1]. All studies were conducted using at least six mice. All experiments were conducted in accordance with the guidelines of the Experimental Animal Research Institute of Jiangnan University.

Individuals aged ≥ 18 years; clinically diagnosed with chronic kidney insufficiency of unknown cause; persistent proteinuria of unknown cause; rapidly progressive deterioration of kidney function with no clear abnormalities in kidney structure or size on imaging and who sign the informed consent for the biopsy procedure. The detailed information about the patients is shown in Table 1. All patients provided written informed consent.

**TABLE 1:** Baseline information for patients.

	BMI > 30 (*n* = 8)	BMI < 23 (*n* = 8)	*p*
Sex (male%)	5 (62.5%)	4 (50%)	
Age	52.63 ± 7.150	53.50 ± 5.099	0.7822
Hypertension (%)	6 (75%)	6 (75%)	
Diabetes (%)	5 (62.5%)	3 (37.5%)	
BMI	32.4 ± 1.149	21.69 ± 1.162	< 0.0001
Systolic blood pressure (mmHg)	142.3 ± 9.881	142.9 ± 14.01	0.9194
Diastolic pressure (mmHg)	90.00 ± 10.74	97.13 ± 7.180	0.1412
Creatinine (μmol/L)	156.9 ± 18.52	125.0 ± 11.84	0.0011
eGFR (mL/min·1.73 m2)	42.4 ± 5.96	45.35 ± 8.26	0.0181
Serum AST (U/L)	37.75 ± 9.036	29.63 ± 7.726	0.0737
Serum ALT (U/L)	39.5 ± 4.106	35.38 ± 3.926	0.0592
Triglycerides level (mmol/L)	3.925 ± 0.6944	1.50 ± 0.2777	< 0.0001
Low‐density lipoprotein (mmol/L)	3.376 ± 0.4173	2.413 ± 0.4549	< 0.0001
Blood glucose	6.78 ± 0.9118	6.67 ± 6985	0.9518
Anti‐hypertension drugs (%)	6 (75%)	6 (75%)	
Anti‐diabetes drugs (%)	5 (62.5%)	3 (37.5%)	

### 2.2 HFD Mouse Model and Senolytic Drug ABT263

Fourteen‐month‐old ApoE^−/−^ and ApoE^−/−^p16^−/−^ mice were fed a high‐fat diet (HFD) (D12108C, SYSEBio‐Tec, Changzhou, China) for 6 months. At the same time, six HFD ApoE^−/−^ mice were injected with the senolytic drug ABT263 by intraperitoneal injection twice a week according to previous research [2]. The HFD contained 60% of calories. The normal diet (ND) contained 20% of calories. After 6 months, kidney tissues and plasma were acquired for analysis.

### 2.3 Western Blot

Protein expressions of p16 (10883‐1‐AP, Proteintech, Wuhan, China), IL‐1β (26048‐1‐AP, Proteintech, Wuhan, China), TNF‐α (17590‐1‐AP, Proteintech, Wuhan, China), IL‐6 (DF6087, Affinity, Jiangsu, China), p19 (10272‐2‐AP, Proteintech, Wuhan, China), p53 (AF0879 Affinity, Jiangsu, China), MMP‐1 (26585‐1‐AP, Proteintech, Wuhan, China), MMP‐3 (17873‐1‐AP, Proteintech, Wuhan, China), ASC (10500‐AP, Proteintech, Wuhan, China), NLRP3 (19771‐1‐AP, Proteintech, Wuhan, China), NLRC4 (AF3580, Affinity, Jiangsu, China), Caspase‐1 (31020‐1‐AP, Proteintech, Wuhan, China), Collagen‐I (14695‐1‐AP, Proteintech, Wuhan, China), a‐SMA (14395‐1‐AP, Proteintech, Wuhan, China), β‐actin (66009‐1‐Ig, Proteintech, Wuhan, China) and GAPDH (60004‐1‐Ig, Proteintech, Wuhan, China) were assessed by the western blot technique. Secondary antibodies were purchased from Proteintech (Wuhan, China). All antibodies were applied according to the manufacturer's instructions.

### 2.4 Immunohistochemistry (IHC) Staining

The experimental process was conducted according to the manufacturer's instructions. Sections were incubated with primary antibodies against Collagen‐I, α‐SMA, IL‐1β, IL‐6, TNF‐α, ASC and NLRP3. Then, the slices were placed in the secondary antibody and processed using the SABC‐POD kit (SA2001, Boster, China). Masson's trichrome staining (Sigma‐Aldrich) was used to detect the degree of fibrosis.

### 2.5 Quantitative Real‐Time Polymerase Chain Reaction (qRT‐PCR)

We used Trizol reagent (Thermo Fisher Scientific) to extract RNA from kidney tissues. According to the manufacturer's instructions (Vazyme), the reverse transcription reaction was performed using the HiScript III first‐strand cDNA synthesis kit. Then, the qRT‐PCR was performed using the FastStart SYBR Green Mix Kit (Sigma‐Aldrich). Determination of GAPDH (CT_control_) and target gene expression levels (CT_target gene_) were performed (Table 2).

**TABLE 2:** Primer for real‐time qPCR assay.

Gene target	Forward sequence (5′‑3′)	Reverse sequence (5′‑3′)
GAPDH	CCCTTAAGAGGGATGCTGCC	TACGGCCAAATCCGTTCACA
Fibronectin	ATGAGAAGCCTGGATCCCCT	GGAAGGGTAACCAGTTGGGG
Vimentin	TTCTCTGGCACGTCTTGACC	CTTTCATACTGCTGGCGCAC
IL‐1β	TGCCACCTTTTGACAGTGATG	TGATGTGCTGCTGCGAGATT
IL‐6	CCCCAATTTCCAATGCTCTCC	CGCACTAGGTTTGCCGAGTA
TNF‐α	GATCGGTCCCCAAAGGGATG	TTTGCTACGACGTGGGCTAC
CXCL2	CCAACCACCAGGCTACAGG	GCGTCACACTCAAGCTCTG
Collagen I	GCTCCTCTTAGGGGCCACT	CCACGTCTCACCATTGGGG
ACTA2	TCTTCCAGCCATCTTTCATTGGGAT	CCTGTTTTGGCTCCCTATGTCT
NLRP3	GGGACCAAATTGAGGGCTTC	TCAACGTCACCAGTCCTCAGA
ASC	GGAGTCGTATGGCTTGGAGC	TGGTCCACAAAGTGTCCTGTT
Caspase‐1	TGCCTGGTCTTGTGACTTGG	GTCACCCTATCAGCAGTGGG
NLRC4	CAGGTCACAGAAGAAGACCTGA	CTCCACACGGTGATGACGAT
MMP9	TCTACAGAGTCTTTGAGTCCG	GGGCTTCCTCTATGATTCAG
MMP3	CAGTCCCTCTATGGAACTCCC	AGGGTGCTGACTGCATCAAA

### 2.6 RNA‐Seq and Bioinformatics Analysis

We extracted RNA from kidney tissue and tested RNA‐seq as previously described. Preparing a cDNA sequencing library used the TruePrep DNA Library Preparation Kit V2 on the Illumina platform and performed 2 × 150 end‐to‐end sequencing. Gene Ontology (GO) and Kyoto Encyclopedia of Genes and Genomes (KEGG) terms were analysed in the Database for Annotation, Visualisation and Integrated Discovery (DAVID). STRING analysis is used to display protein interaction networks, whereas PANTHER analysis is used to display molecular functions and gene pathways.

### 2.7 Determination of Serum Urea Nitrogen and Serum Creatinine

Mice were anaesthetised by intraperitoneal injection of 3% pentobarbital sodium (40 mg/kg) and drew blood from the mouse heart using a 1 mL syringe. Then, we detected the level of serum urea nitrogen (SUN) (C013‐2 SUN test kit) and serum creatinine (SCr) (C011 Cr detection kit) in serum according to the manufacturer's instructions (Nanjing Jiancheng Institute of Bioengineering, China) [3].

### 2.8 Metabolite Annotation

Gene Ontology (GO) and Kyoto Encyclopedia of Genes and Genomes (KEGG) terms were analysed in Database for Annotation, Visualisation and Integrated Discovery (DAVID). STRING analysis was used to show protein interaction networks, and PANTHER analysis was used to show molecular functions and gene pathways.

### 2.9 Statistical Analysis

Data are expressed as mean ± SEM and analysed by SPSS (Version 19.0; SPSS Inc., Chicago, IL, USA) or GraphPad Prism software (Version 6.02). For two groups comparison, data were analysed with two‐tailed unpaired *t*‐tests. For multiple group comparisons, data were analysed using one‐way ANOVA with LSD test. *p* < 0.05 was considered statistically significant.

## 3 Results

### 3.1 p16 Was Over‐Expression in Obesity‐Associated Kidney Fibrosis

To clarify p16 and its role in obesity‐associated kidney fibrosis, we treated ApoE knockout (ApoE^−/−^) mice with a high‐fat diet (HFD) (containing 60% calories) for 6 months and recorded the weights of the mice every week. We found that an HFD could significantly increase the weight of the mice. We then collected the kidneys for further immunoblotting analysis, and the results revealed that, compared with the normal diet, the HFD increased the expression level of p16INK4a in the kidney (Figure S1A,B). Additionally, we confirmed the expression level of p16INK4a via qPCR assay and reached the same conclusion (Figure S1C). These results revealed that an HFD could induce the overexpression of p16INK4a in the kidney after 6 months.

### 3.2 p16 Knockout Alleviated the Fibrotic Changes in HFD‐Induced Kidneys

**FIGURE 1:**
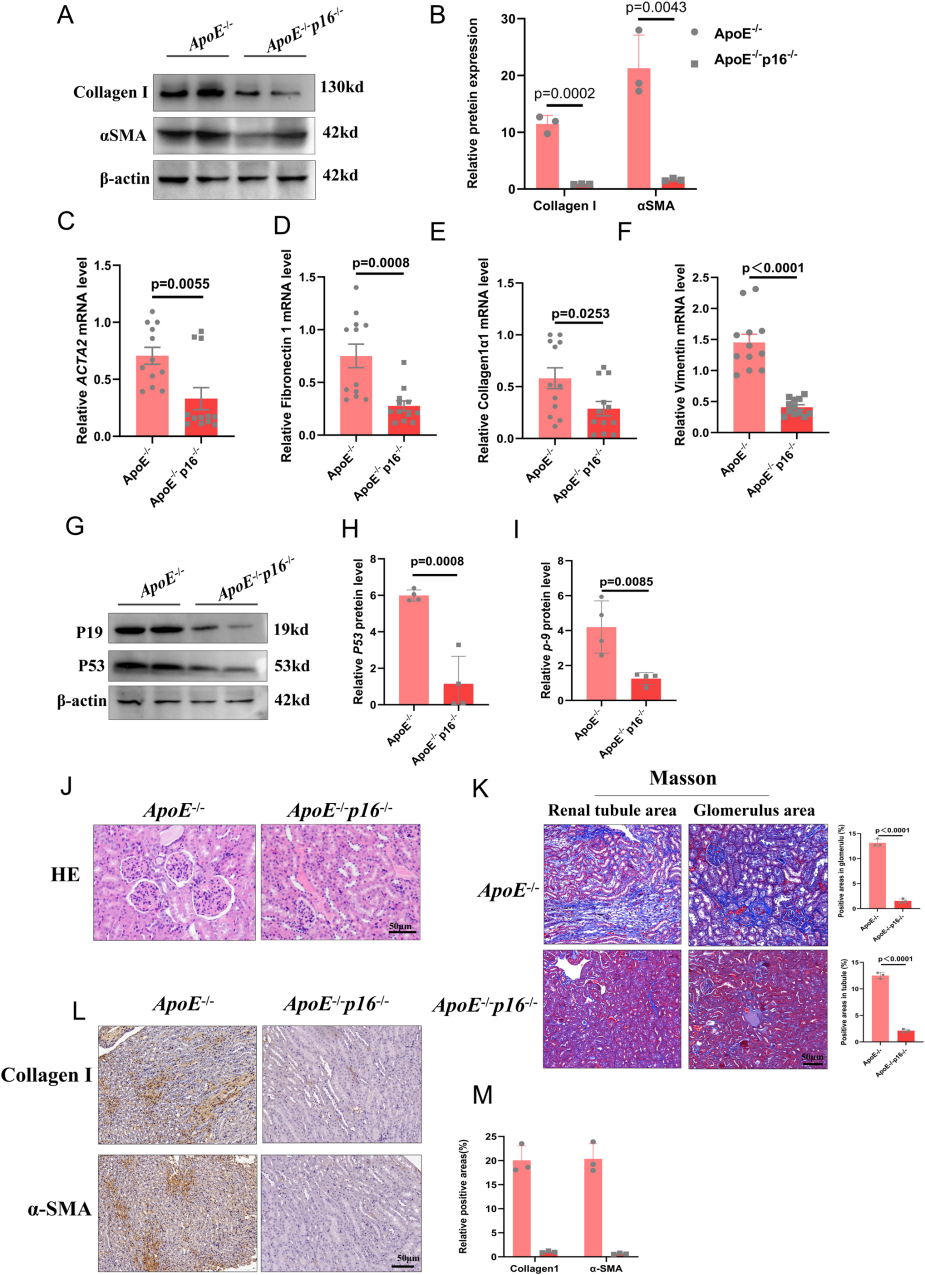
p16 knockout (p16^−/−^) mice ameliorate high‐fat diet‐induced renal fibrosis. Fourteen‐month‐old double knockout (KO) ApoE^−/−^p16^−/−^ (DKO) mice and ApoE^−/−^ littermates were fed a high‐fat diet (HFD) for 6 months and obtained renal tissues for further analysis. (A, B) expression levels and statistical figures of α‐SMA and Collagen I in renal tissues from 20‐month ApoE^−/−^ mice and DKO mice induced by HFD were detected by western blotting (*n* = 4); (C–F) Fluorescence quantitative PCR experiments were performed to detect the transcription levels of ACTA2, Fibronectin1, Collagen I‐1α and Vimentin in the renal tissues (*n* = 12); (G–I) expression levels and statistical figures of p19 and p53 in renal tissues from 20‐month ApoE^−/−^ mice and DKO mice induced by HFD were detected by western blotting (*n* = 4); (J) H&E staining showing inflammatory infiltration of renal from 20 months ApoE^−/−^ mice and DKO mice induced by HFD (*n* = 3); (K) representative images of Masson staining to assess renal tubule and glomerulus collagen deposition from 20‐month ApoE^−/−^ mice and DKO mice induced by HFD (*n* = 3); (L, M) expression levels and statistical figures of Collagen I and α‐SMA in renal tissues from 20‐month ApoE^−/−^ mice and DKO mice detected by immunohistochemical staining (*n* = 3); values are mean ± SEM, *p* < 0.05 compared with ApoE^−/−^ mice.

We found that p16 was overexpressed in HFD‐induced kidneys. We then hypothesized that p16 plays a critical role in HFD‐induced kidney changes. We subsequently used 14‐month‐old ApoE^−/−^ p16^−/−^ mice and ApoE^−/−^ mice, which were fed an HFD for 6 months, and collected the tissues. Our research revealed that p16 knockout decreased the expression of multiple profibrotic proteins (α‐SMA and collagen I) in HFD‐induced kidneys (Figure 1A,B). To further test this conclusion, we detected the expression levels of profibrotic genes in HFD‐induced kidneys and found that, compared with those in the ApoE^−/−^ group, p16 knockout decreased the expression levels of *ACTA2* (Figure 1C), *fibronectin 1* (Figure 1D), *Col1α1* (Figure 1E) and *vimentin* (Figure 1F). Additionally, we found that p16 inhibited the expression of p19 and p53 in HFD‐induced kidneys (Figure 1G–I). HE staining (Figure 1J) revealed antifibrotic effects after p16 knockout. Furthermore, the results of Masson staining (Figure 1K) indicated that, compared with those in the ApoE^−/−^ group, significant antifibrotic pathological changes in the glomerulus and renal tubule areas were observed after p16 deletion. In addition, p16 deletion inhibited collagen I and α‐SMA protein expression in HFD‐induced kidneys (Figure 1L,M). These results indicated that p16 knockout alleviated the fibrotic changes in HFD‐induced kidneys.

### 3.3 p16 Knockout Ameliorated the Inflammatory Phenotype of Hfd‐Induced Kidneys

**FIGURE 2:**
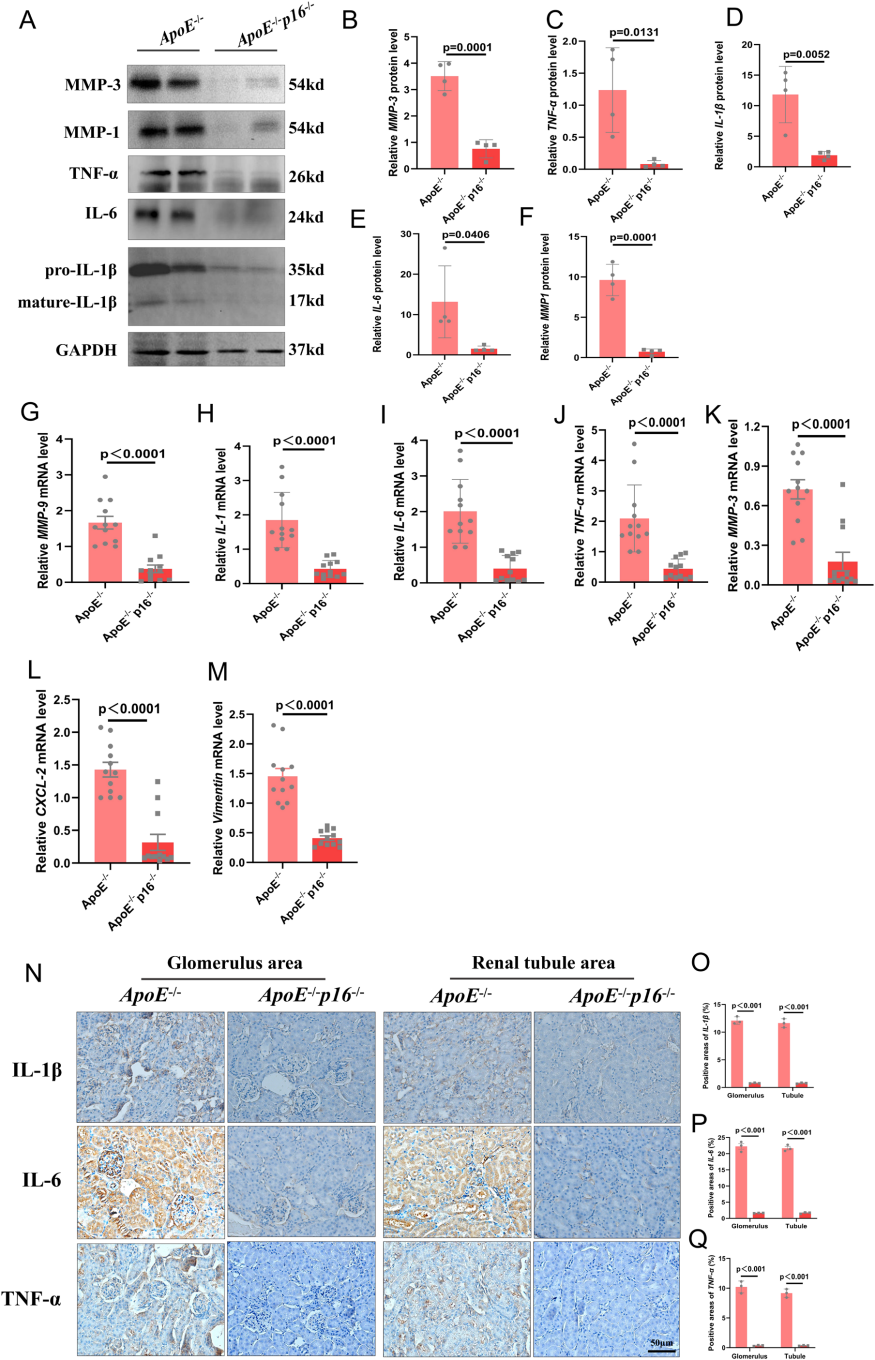
p16 knockout (p16^−/−^) mice ameliorate high‐fat diet (HFD)‐induced renal inflammation of ApoE^−/−^ mice. (A–F) expression levels and statistical figures of MMP1, MMP3, IL‐1β, TNF‐α and IL‐6 in renal tissues from 20‐month ApoE^−/−^ mice and DKO mice induced by HFD were detected by western blotting (*n* = 4); (G–M) Fluorescence quantitative PCR experiments were performed to detect the transcription levels of MMP3, MMP9, IL‐1β, TNF‐α, IL‐6, CXCL‐2 and Vimentin in the renal tissues (*n* = 12); values are mean ± SEM, *p* < 0.05 compared with ApoE^−/−^ mice; (N–P) Statistical analysis of IL‐1β and IL‐6 in renal tissues from 20‐month ApoE^−/−^ mice and DKO mice detected by immunohistochemical staining (*n* = 3); values are mean ± SEM, *p* < 0.05 compared with ApoE^−/−^ mice; (Q) statistical analysis of TNF‐α in renal tissues from 20‐month ApoE^−/−^ mice and DKO mice detected by immunohistochemical staining (*n* = 3); values are mean ± SEM, *p* < 0.05 compared with ApoE^−/−^ mice.

We then assessed the expression levels of multiple proinflammatory cytokines and found that p16 knockout inhibited the expression levels of MMP1, MMP3, IL‐1β, TNF‐α and IL‐6 in HFD‐induced kidneys (Figure 2A–F). We then evaluated the results via a qPCR assay and found that the mRNA levels of various inflammatory cytokines were decreased (Figure 2G–M). Further validation via renal tissue immunohistochemistry and statistical analysis confirmed that the expression levels of IL‐1β, TNF‐α and IL‐6 in the glomerulus and renal tubule areas were significantly lower in p16KO mice than in ApoE^−/−^ mice (Figure 2N–P). Additionally, the statistical analysis confirmed that the expression levels of TNF‐α in the glomerulus and renal tubule areas were significantly lower in p16KO mice than in ApoE^−/−^ mice (Figure 2Q).

### 3.4 p16 Knockout Inhibited Activation of the Nlrp3 Inflammasome Pathway in Hfd‐Induced Kidneys

**FIGURE 3:**
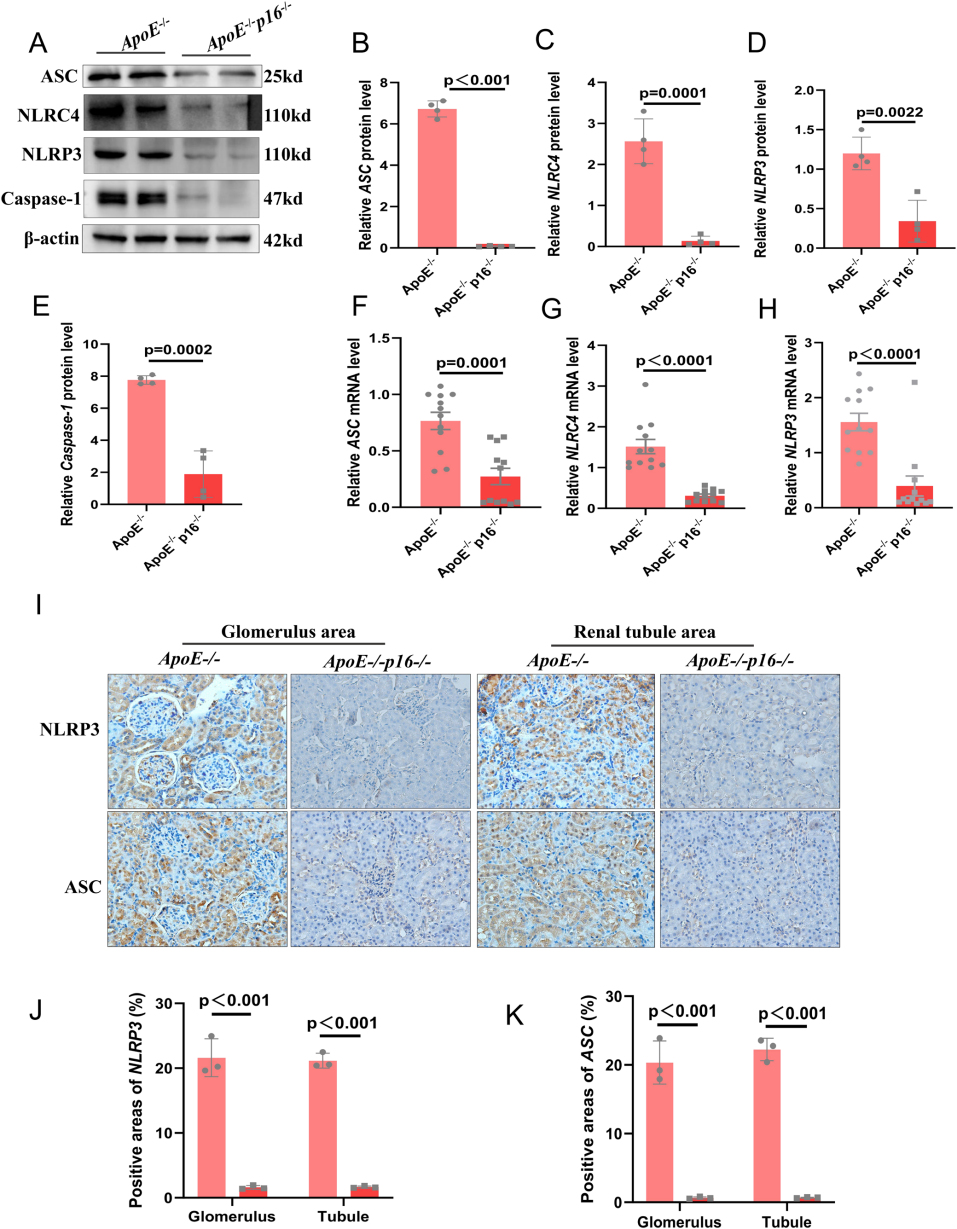
p16 knockout (p16^−/−^) ameliorates the NLRP3 inflammasome pathway. (A–E) Expression levels and statistical figures of ASC, Caspase‐1, NLRC4 and NLRP3 in renal tissues from 20 months ApoE^−/−^ mice and DKO mice induced by HFD were detected by western blotting (*n* = 4); (F–H) Fluorescence quantitative PCR experiments were performed to detect the transcription levels of ASC, NLRC4 and NLRP3 in the renal tissues (*n* = 12); (I, K) expression levels and statistical analysis of ASC and NLRP3 in renal tissues from 20 months ApoE^−/−^ mice and DKO mice detected by immunohistochemical staining (*n* = 3); values are mean ± SEM, *p* < 0.05 compared with ApoE^−/−^ mice.

We then detected the expression levels of proteins associated with the NLRP3 inflammasome pathway and found that p16 knockout inhibited the expression levels of ASC, NLRP3, NLRC4 and Caspase‐1 in HFD‐induced kidneys (Figure 3A–D). We then evaluated the results via a qPCR assay and found that the mRNA levels of various inflammatory cytokines were decreased (Figure 3E–H). Further verification via immunohistochemical analysis of kidney tissue confirmed that the expression levels of ASC and NLRP3 were significantly lower in p16KO mice than in ApoE^−/−^ mice in the glomerulus and renal tubule areas (Figure 3I–K).

### 3.5 ABT263 Administration Alleviated Fibrosis and the Inflammatory Phenotype of Hfd‐Induced Kidneys

**FIGURE 4:**
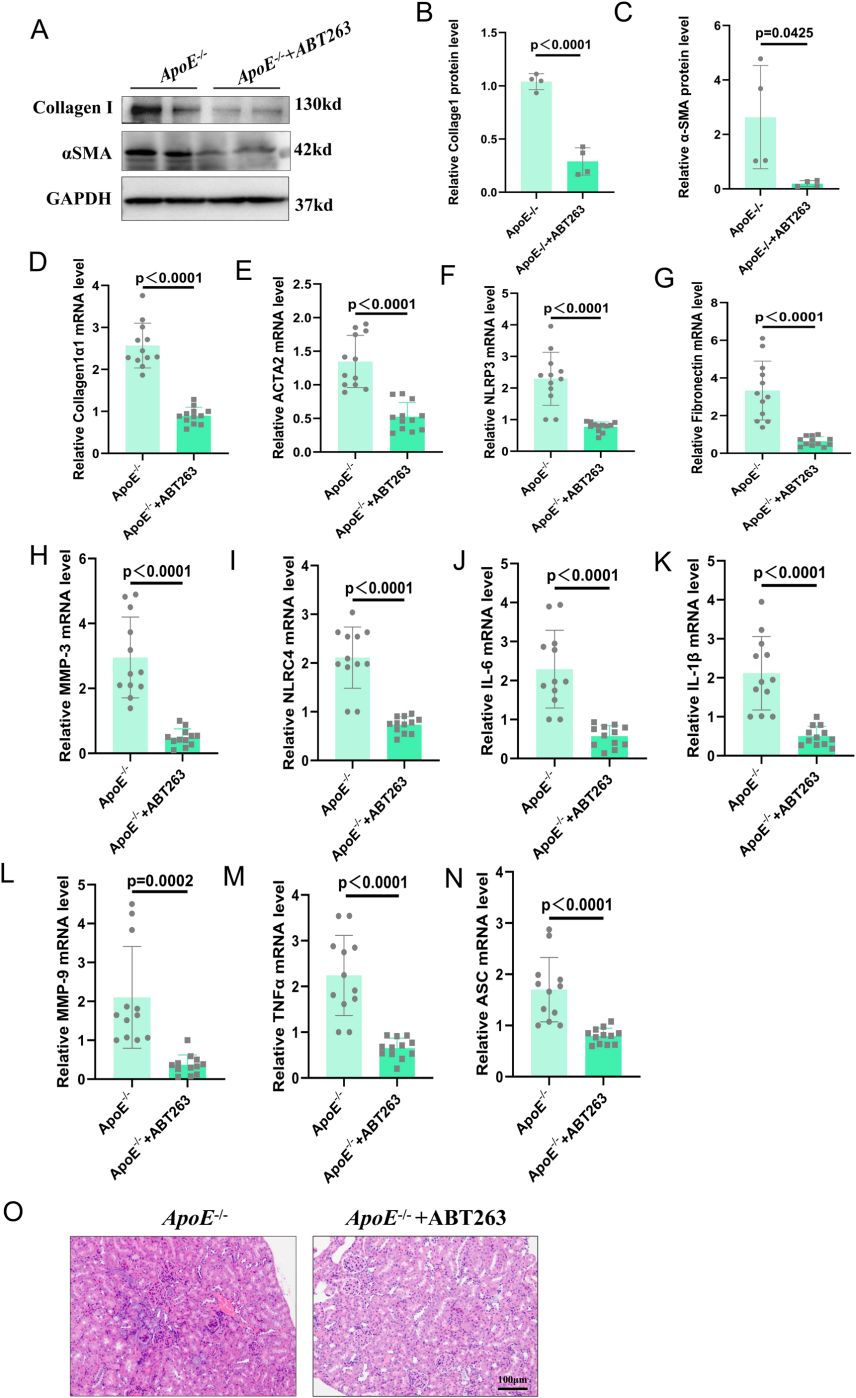
ABT263 treatment inhibits HFD‐induced renal fibrosis in ApoE^−/−^ mice. (A–C) Expression levels and statistical figures of α‐SMA and Collagen I in renal tissues from 20‐month ApoE^−/−^ mice and ApoE^−/−^ treated with ABT263 mice induced by HFD were detected by western blotting (*n* = 4); (D–N) Fluorescence quantitative PCR experiments were performed to detect the transcription levels of ASC, NLRC4, NLRP3, MMP1, MMP3, IL‐1β, TNF‐α, IL‐6, Collagen I 1α, ACTA2, and Fibronectin in the renal tissues (*n* = 12); (O) H&E staining showing inflammatory infiltration of renal tissues from 20‐month ApoE^−/−^ mice and ApoE^−/−^ treated with ABT263 mice induced by HFD (*n* = 3); values are mean ± SEM, *p* < 0.05 compared with ApoE^−/−^ mice.

To further confirm the antifibrotic and anti‐inflammatory effects in HFD‐induced kidneys, we treated HFD‐induced mice with ABT263 and found that the expression levels of collagen I and α‐SMA were significantly decreased after ABT263 administration (Figure 4A–C). Additionally, ABT263 administration decreased the expression levels of *ACTA2*, *Col1α1* and fibronectin in HFD‐induced kidneys. The expression levels of various cell senescence‐associated proteins (p53 and p16) were measured, revealing the inhibitory effect of ABT263 treatment (Figure S2A,S2B). Masson staining revealed that ABT263 administration alleviated kidney fibrosis in ApoE^−/−^ mice in the glomerular and renal tubule areas (Figure S3).

To further clarify the anti‐inflammatory effect of ABT263, we concluded that ABT263 administration inhibited the mRNA expression levels of IL‐1β, TNF‐α, IL‐6, MMP3, MMP9, NLRP3 and ASC in ApoE^−/−^ mice fed an HFD (Figure 4G–N). HE staining revealed that ABT263 alleviated the inflammatory response and fibrosis in the glomerulus and renal tubule areas of ApoE^−/−^ mice (Figure 4O).

**FIGURE 5:**
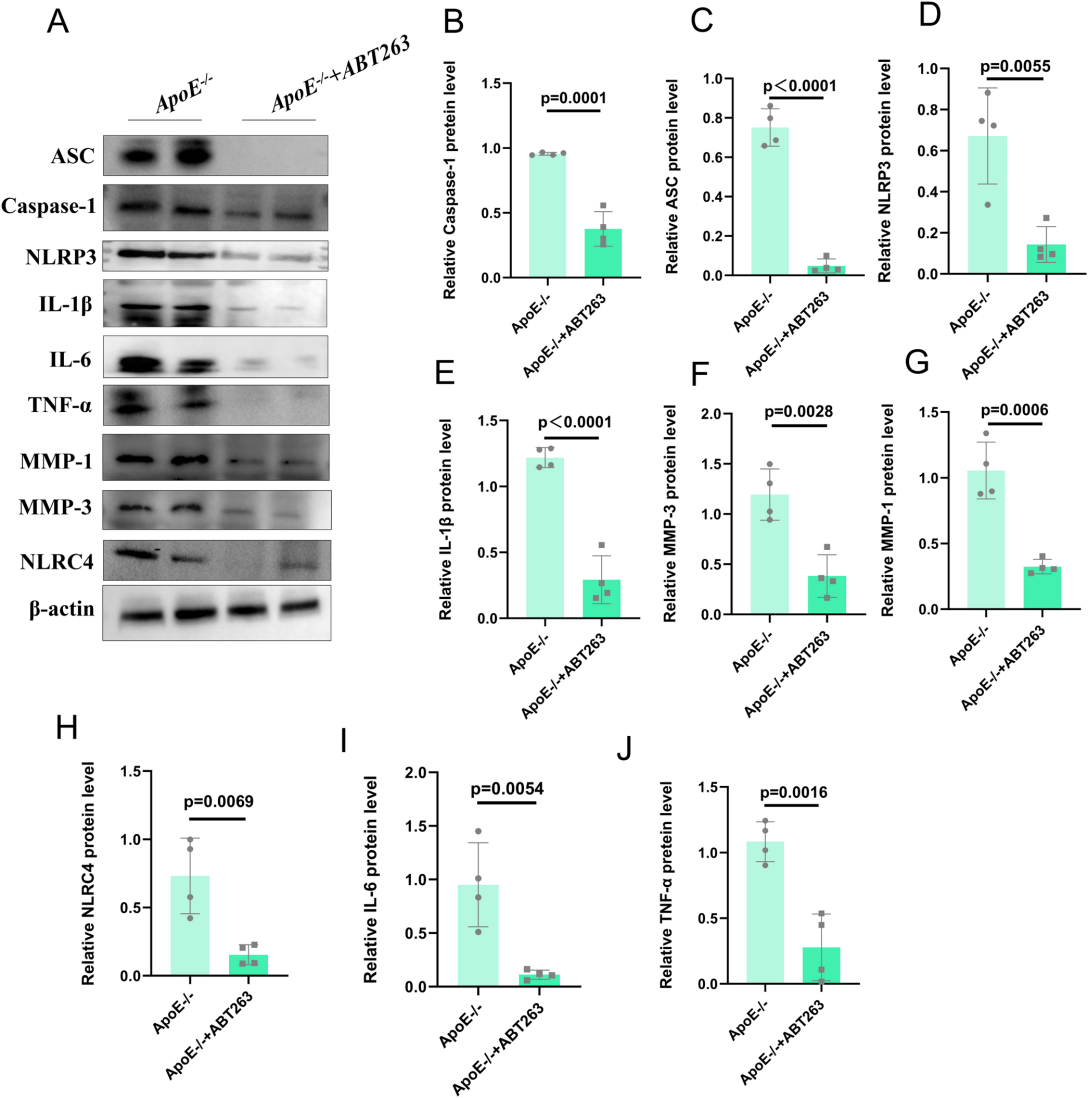
ABT263 treatment inhibits high‐fat diet (HFD)‐induced renal inflammation of ApoE^−/−^ mice. (A–J) Expression levels and statistical figures of ASC, NLRC4, NLRP3, caspase‐1, MMP1, MMP3, IL‐1β, TNF‐α and IL‐6 in renal tissues from 20‐month‐old ApoE^−/−^ mice and ApoE mice^−/−^ treated with ABT263 induced by HFD were detected by western blotting (*n* = 4); values are mean ± SEM, *p* < 0.05 compared with ApoE^−/−^ mice.

We then assessed the expression levels of proteins associated with the NLRP3 inflammasome pathway and found that p16 knockout inhibited the expression levels of ASC, NLRP3, NLRC4 and Caspase‐1 in HFD‐induced kidneys (Figure 5A–D). We then evaluated the anti‐inflammatory effect of ABT263 via Western blotting and found that the protein levels of various inflammatory cytokines were decreased after treatment with ABT263 (Figure 5E–J).

Overall, we reported that ABT263 administration alleviated fibrosis and the inflammatory phenotype of HFD‐induced kidneys.

### 3.6 p16 Knockout Remodelled the Metabolic Shift in Hfd‐Induced Kidneys

**FIGURE 6:**
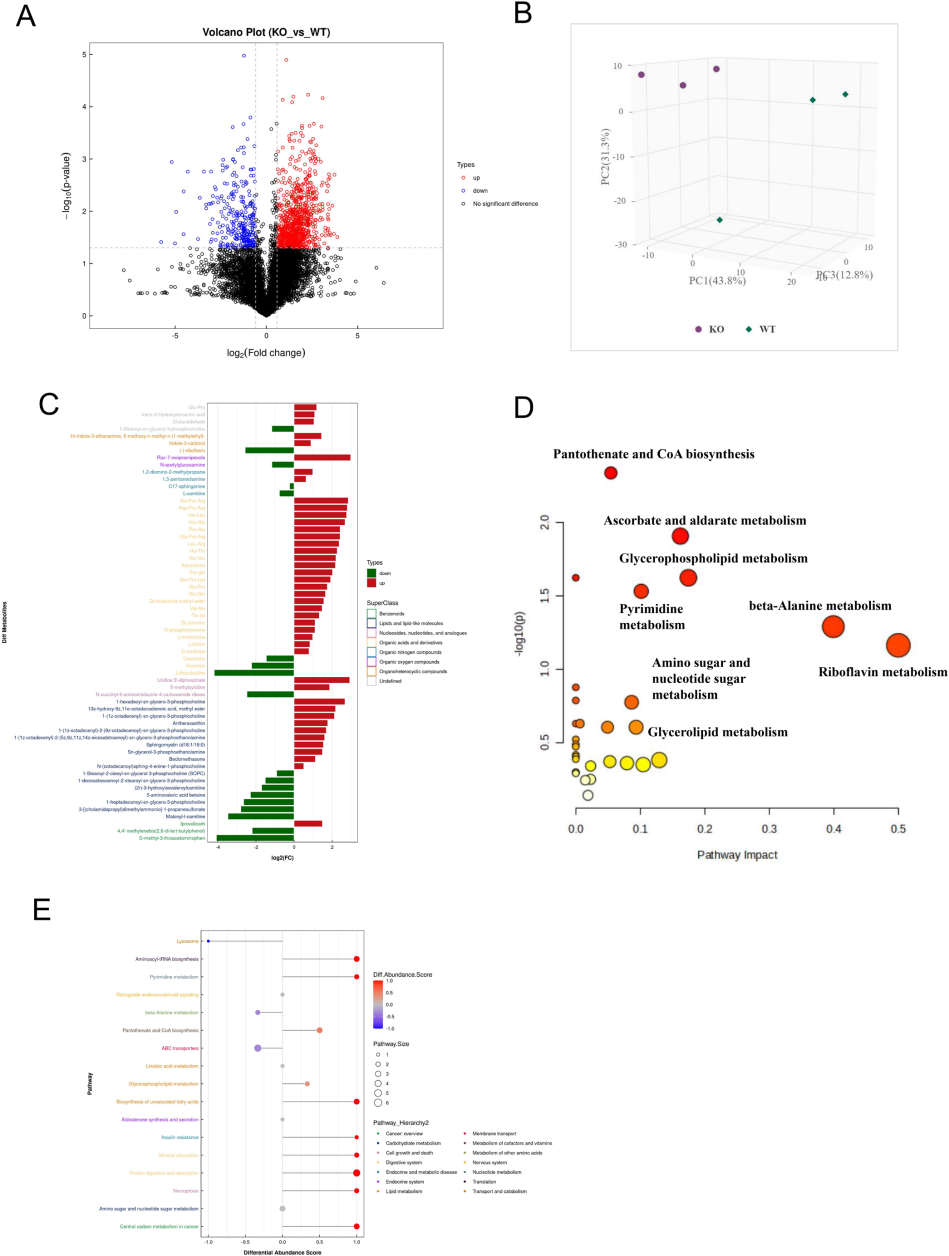
p16 knockout remodelled metabolic shift in HFD induced kidney. (A) Metabolomics analysis on the kidney tissues (*n* = 3) identified statistically significant (*p* < 0.05) differential metabolites in positive‐ion mode, which were presented as a volcano plot: Fold change from p16KO (i.p.) mice versus WT mice; (B) Pie chart of metabolite classification in positive‐ion mode; (C) box plot showed the top differential metabolites in positive‐ion mode; (D) scatter plots showed the matched pathways enriched by the KEGG library according to *p*‐values from pathway enrichment analysis (*y*‐axis) and pathway impact values from pathway topology analysis (*x*‐axis) via MetaboAnalyst 5.0; (E) scatter plots showed the matched enrichment pathway analysis (*y*‐axis) and differential abundance score analysis by the SMPDB library (*x*‐axis) via MetaboAnalyst 5.0.

**FIGURE 7:**
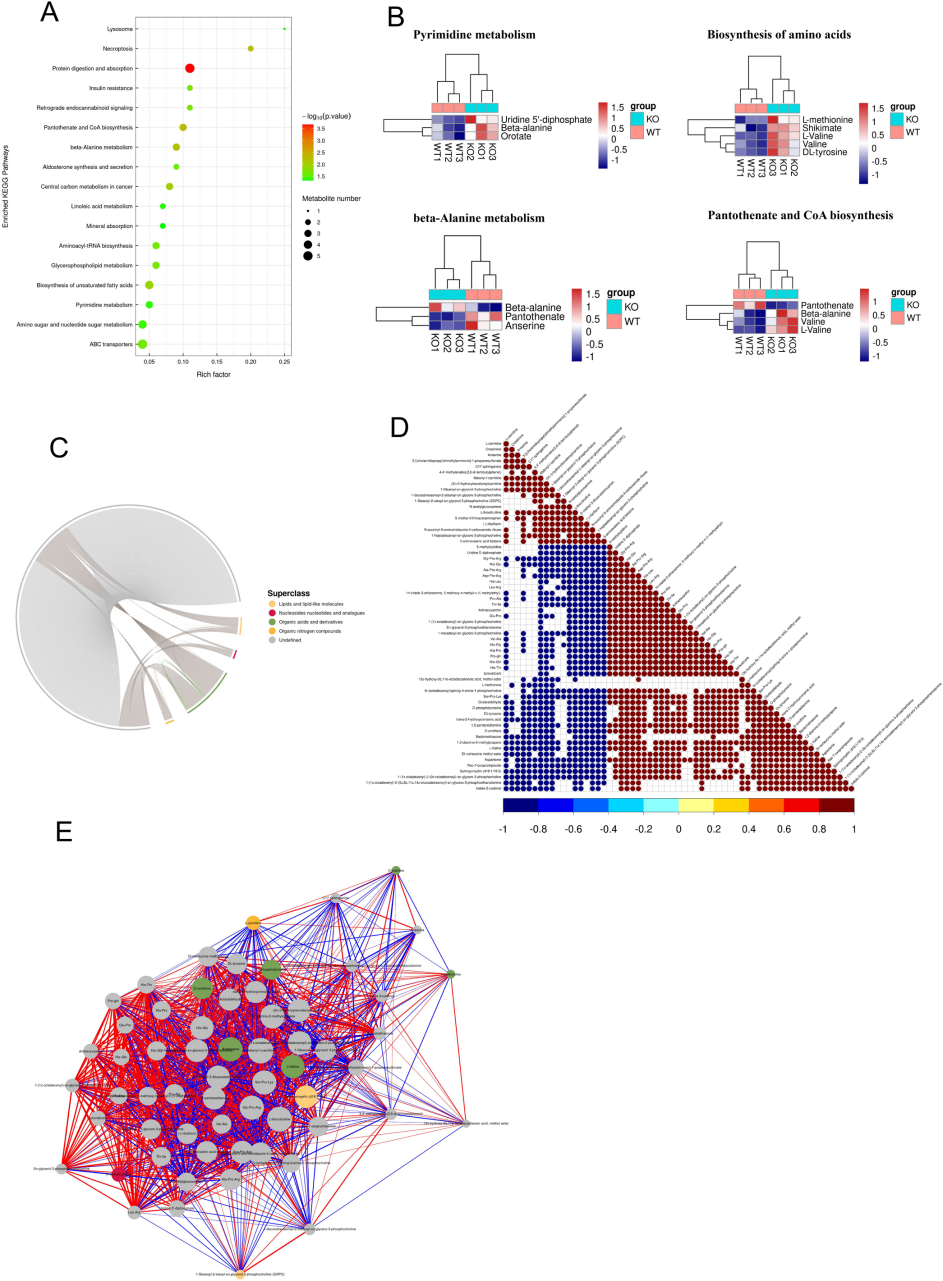
p16 knockout regulated the Pyrimidine metabolism and beta‐Alanine metabolism pathway in kidneys. (A) KEGG pathway annotation. The horizontal coordinate represents the number of metabolites and the vertical coordinate represents the annotated KEGG pathway; (B) Heatmap showed differential metabolites identified in positive‐ion mode in Pyrimidine metabolism, Biosynthesis of amino acids, beta‐Alanine metabolism, and Pantothenate and CoA biosynthesis; (C) Superclass analysis of metabolites detected via Metabolomics; (D) Spearman Correlation analysis diagram of differential metabolites. The highest correlation is 1, which is a complete positive correlation (red); the lowest correlation is −1, which is a complete negative correlation (blue); the part without colour indicates *p*‐value > 0.05; (E) Pearson correlation analysis of the differential metabolites with significantly changed faecal metabolites (in positive‐ion mode) using a network diagram.

To further clarify the role of p16 in the metabolic shift of the kidney in ApoE^−/−^ mice, after metabolomic sequencing of mouse kidney tissue, We found that metabolites associated with lipid and lipid‐like molecules and organic acids and derivatives were mainly detected (Figure S4A). Partial least squares discrimination analysis (Figure S4B) shows that Metabolomics data can better reflect and describe the sample differences. We discovered significant differences in renal metabolism between p16 knockout and wild‐type (WT) mice through principal component analysis (PCA). These findings suggest that the p16 protein plays a role in the metabolic changes observed in the kidneys of mice subjected to a high‐fat diet (Figure 6A,B). Further analysis using the MetaboAnalyst platform revealed notable enrichment in several pathways, including pantothenate and CoA biosynthesis, ascorbate and aldarate metabolism, beta‐alanine metabolism and amino sugar and nucleotide sugar metabolism (Figure 6D). Also, SMPDB analysis shown notable enrichment in several pathways, including Glycerophospholipid metabolism, ABC transporter, necroptosis and pyrimidine metabolism (Figure 6E). KEGG pathway analysis revealed that p16 knockout could induce the activation of linoleic acid metabolism, necroptosis, insulin resistance, etc., in ApoE^−/−^ mice after they were fed an HFD, and also we found that the differential Metabolites mainly were organic acids and derivatives and organic nitrogen compounds (Figure 7A,C). The detailed expression levels of differential Metabolites associated with pyrimidine metabolism and β‐Alanine metabolism are shown in Figure S4C. In addition, p16 knockout increased the expression levels of metabolites associated with pyrimidine metabolism, amino acid biosynthesis, beta‐alanine metabolism and pantothenate and CoA biosynthesis (Figure 7B). We also found that p16 knockout remodelled the metabolic shift in HFD‐induced kidneys. Spearman analysis (Figure 7D) and Pearson analysis (Figure 7E) show the correlation analysis between differential metabolites after p16 knockout in kidneys.

### 3.7 p16 is Overexpressed in the Kidneys of Chronic Kidney Disease Patients With Long‐Term Hyperlipidemia

**FIGURE 8:**
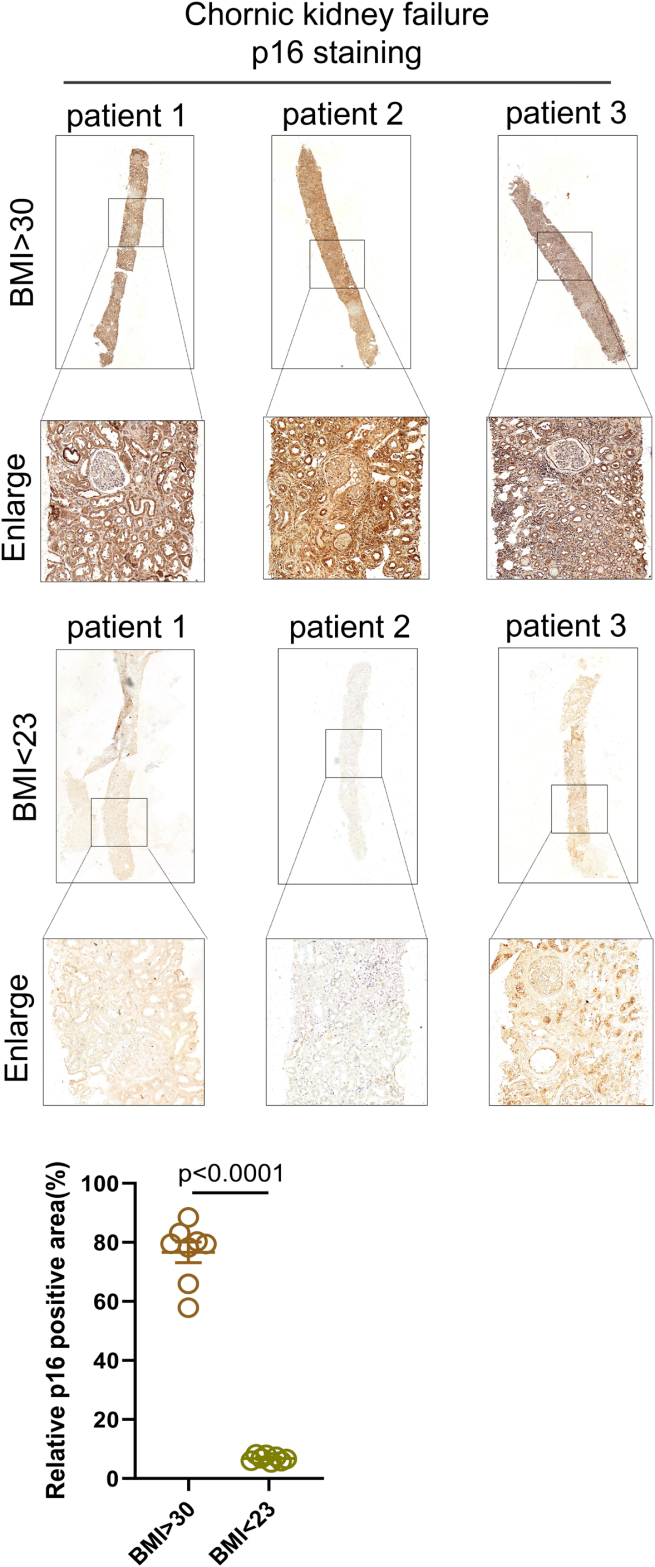
p16 is highly expressed in renal tissue of obese patients with chronic renal failure. (A, B) Expression levels and statistical analysis of p16 in renal tissues from chronic renal failure patients with BMI > 30 and BMI < 23 detected by immunohistochemical staining (*n* = 3); values are mean ± SEM.

To further confirm this conclusion in clinical patients, we collected clinical samples from patients with chronic kidney disease with or without hyperlipidemia. We then assessed the expression levels of p16 in kidneys from clinical patients via immunohistochemistry. We found that p16 was overexpressed in chronic kidney disease patients with long‐term hyperlipidemia (> 15 years) but not in chronic kidney disease patients without hyperlipidemia (Figure 8A,B).

## 4 Discussion

In this study, we observed elevated expression levels of p16 in the kidneys of mice subjected to a high‐fat diet (HFD). The knockout of p16 was found to inhibit the activation of the NLRP3 inflammasome pathway, thereby mitigating the inflammatory phenotype induced by the HFD in the kidneys. Furthermore, p16 knockout reduced the levels of several proinflammatory biomarkers, including IL‐1β, IL‐6 and TNF‐α. Metabolomic analysis indicated that p16 knockout activated pathways associated with linoleic acid metabolism, necroptosis, and insulin resistance in ApoE^−/−^ mice following HFD exposure. Additionally, the administration of ABT263 inhibited the NLRP3 inflammasome pathway and reduced the expression levels of proinflammatory biomarkers (such as IL‐1β, IL‐6 and TNF‐α), thereby alleviating the inflammatory phenotype of HFD‐induced kidney injury in ApoE^−/−^ mice.

The inflammatory microenvironment established by senescent cells plays a critical role in the pathogenesis of kidney disease [4, 5]. Our previous studies using a premature senescence mouse model revealed a marked proinflammatory microenvironment accompanied by an accumulation of senescent cells [6], which was closely linked to oxidative stress and the overactivation of DNA damage response pathways [7, 8]. Notably, excessive accumulation of senescent cells was also observed in acute kidney injury models. p16 is frequently associated with cellular senescence and is implicated in kidney ageing and damage [9, 10]. For example, p16 overexpression has been reported in renal tubular epithelial cells in diabetic nephropathy [11, 12, 13]. p16 knockout was shown to reverse renal senescence in Bmi‐1‐deficient mice [6]. Furthermore, our research demonstrated that p16 knockout improved outcomes in acute kidney injury models induced by contrast agents and glycerin. Despite these findings, the specific mechanisms by which p16 modulates kidney changes following HFD exposure remain inadequately explored. To address this, we established a chronic kidney injury model in ApoE^−/−^ mice through HFD feeding. Our data corroborate previous findings by showing that p16 expression levels are elevated in response to an HFD. Importantly, p16 knockout was found to enhance renal function by reducing kidney fibrosis in ApoE^−/−^ mice. Additionally, p16 deletion decreased the levels of various pro‐inflammatory biomarkers (including IL‐1β, IL‐6 and TNF‐α). We also observed that ABT263 administration reduced pro‐inflammatory biomarker levels and alleviated kidney fibrosis.

The NLRP3 inflammasome is a central component of the innate immune system and is essential for detecting harmful stimuli and orchestrating an inflammatory response [14, 15]. NLRP3 is activated by a range of stimuli, including metabolic disturbances, damage‐associated molecular patterns (DAMPs) and pathogen‐associated molecular patterns (PAMPs) [16]. Upon activation, NLRP3 recruits the adaptor protein ASC via its pyrin domain, leading to the formation of a large protein complex that recruits and activates caspase‐1. Activated caspase‐1 then cleaves pro‐IL‐1β and pro‐IL‐18 into their active forms, IL‐1β and IL‐18, respectively [17, 18]. These cytokines are subsequently released into the extracellular space, where they promote inflammation and attract immune cells. Our previous research demonstrated that p16 deletion inhibits NLRP3 inflammasome activation through interactions with SGK1 [2]. Conversely, p16 overexpression has been associated with NLRP3 inflammasome activation in acute kidney injury models via oxidative stress regulation. Previous studies have shown that factors such as hypertension, diabetes and infections can induce stress in kidney cells, leading to NLRP3 activation [19, 20].

The mechanisms underlying metabolic remodelling in chronic kidney disease are intricate. Abnormal fatty acid metabolism can impair mitochondrial function, resulting in diminished energy production and heightened oxidative stress, which significantly contribute to renal fibrosis [21, 22]. Additionally, pyrimidine metabolism—which involves the synthesis and degradation of pyrimidine nucleotides essential for DNA and RNA synthesis—plays a role in the progression of renal fibrosis [22]. Disruptions in pyrimidine metabolism can affect the cellular energy balance, signalling pathways and DNA damage response, thereby impacting cell function and survival under fibrotic conditions [23]. Our study demonstrated that p16 knockout significantly enhances pyrimidine metabolism in renal cells. Disruptions in pyrimidine metabolism can lead to increased mutagenesis and cellular dysfunction, thereby exacerbating oxidative stress and contributing to the secretion of senescence‐associated secretory phenotype factors [24].

β‐Alanine metabolism, though less frequently discussed, plays a role in several important metabolic processes [25]. β‐Alanine is involved in the synthesis of carnosine, an antioxidant that neutralises reactive oxygen species (ROS) and reduces oxidative stress, a major contributor to ageing [26, 27]. β‐Alanine supplementation can increase carnosine levels, which may benefit ageing individuals by improving muscle function, cognitive health and oxidative stress management [28, 29]. Research suggests that carnosine may have therapeutic potential in neurodegenerative diseases such as Alzheimer's disease and Parkinson's disease by mitigating oxidative damage and enhancing neuronal health [30, 31]. Our study indicates that p16 knockout promotes β‐alanine metabolism in renal cells, suggesting that this metabolic remodelling may be a key mechanism by which p16 knockout inhibits inflammatory ageing.

As described in our study, p16 knockout ameliorated renal fibrosis in obese mice. The pathogenesis of obesity‐related renal fibrosis is complex, and metabolic remodelling is the key cause. Studies have shown that abnormal lipid metabolism occurs in obesity‐related renal fibrosis due to the excessive production of inflammatory cytokines such as tumour necrosis factor and interleukin‐6 (IL‐6) in kidneys [32]. Additionally, hyperlipidemia leads to inflammation, resulting in cellular dysfunction and pathological alterations in the renal glomeruli [33]. Previous research indicated that excessive activation of the NF‐κB pathway, production of oxygen free radicals, and secretion of inflammatory cytokines have been implicated in obesity‐related renal fibrosis and obesity‐related nephropathy [34]. The activation of these pathways is closely related to obesity‐related metabolic pathway alterations. Studies have shown that β‐Alanine is involved in the synthesis of carnosine, an antioxidant that neutralises reactive oxygen species (ROS) and reduces oxidative stress and finally inhibits the secretion of inflammatory cytokines and down‐stream NF‐κB pathway [26, 27]. Our study shows that p16 knockout could activate the β‐Alanine metabolic pathway in renal cells, thus inducing the anti‐inflammatory and anti‐fibrotic effect in ApoE knockout mice. Luo et al. [35] showed that the metabolite Ureidopropionic acid, originating from β‐alanine metabolism, could inhibit the inflammatory response in Macrophages. β‐alanine supplementation contributed to the protective effects on cardiometabolic health and cardiovascular, hepatic and renal function in adults with overweight and obesity [36]. Furthermore, previous research showed that abnormal pyrimidine metabolism, contributing to the increasing level of nucleotides in glomerular uracil nucleotides, led to the chronic expansion of the uracil nucleotide pool and was associated with the thickening of the glomerular basement membrane, thus finally aggravating chronic kidney diseases [37]. Song et al. [38] showed that metabolic disorders of pyrimidine metabolism in diabetic nephropathy led to the activation of the NF‐κB pathway. In addition to the regulatory role of pyrimidine metabolism in renal fibrosis, abnormal pyrimidine metabolism aggravated the process of nonalcoholic fatty liver disease and liver fibrosis. Restoring the pyrimidine metabolism pathway via Sodium‐glucose cotransporter 2 (SGLT2) inhibitors could alleviate hepatic changes in nonalcoholic fatty liver disease [39]. In this research, we found that p16 knockout could inhibit the over‐activation of pyrimidine metabolism in the kidney, thus reducing the levels of multiple pro‐inflammatory cytokines to alleviate renal fibrosis. Our study demonstrated that p16 knockout may alter β‐Alanine metabolism and pyrimidine metabolism in the kidney, which may suppress chronic renal inflammation, inhibit the NF‐κB pathway, and ultimately alleviate obesity‐related renal fibrosis.

In conclusion, our study elucidates the critical role of p16 in exacerbating kidney inflammation and dysfunction associated with HFD‐induced chronic kidney injury in ApoE^−/−^ mice. We found that p16 overexpression activated the NLRP3 inflammasome pathway, leading to elevated levels of proinflammatory biomarkers and subsequent renal damage. Importantly, p16 knockout mitigates these effects by inhibiting the NLRP3 inflammasome, reducing cytokine levels and improving renal function. Our findings also highlight the impact of p16 deletion on metabolic pathways, such as linoleic acid metabolism and pyrimidine metabolism, suggesting that p16 knockout induces beneficial metabolic remodelling. Additionally, ABT263 administration reinforces these effects by suppressing inflammation and fibrosis in the kidneys. Collectively, these results underscore the potential of targeting p16 and related pathways as a strategy for mitigating the inflammation and renal damage associated with high‐fat diets. Future research should explore these mechanisms and evaluate potential therapeutic strategies for chronic kidney disease.

## Author Contributions

**Qian Liu:** conceptualization (equal), data curation (equal), formal analysis (equal), investigation (equal), software (equal), writing – original draft (equal). **Fen Wang:** conceptualization (equal), formal analysis (equal), funding acquisition (equal), investigation (equal), project administration (equal), validation (equal), writing – original draft (equal), writing – review and editing (equal). **Yuan Du:** conceptualization (equal), formal analysis (equal), investigation (equal), software (equal), supervision (equal), writing – original draft (equal). **Yankui Liu:** data curation (equal), investigation (equal), methodology (equal). **Zhixuan Zhang:** conceptualization (equal), data curation (equal), investigation (supporting), software (supporting). **Xiaodong Zhang:** formal analysis (equal), investigation (supporting), methodology (supporting), visualization (equal). **Jianwei Li:** formal analysis (equal), software (equal), validation (equal), visualization (supporting). **Guangyi Huang:** data curation (equal), formal analysis (equal), investigation (supporting), software (supporting). **Fengqi Liu:** investigation (supporting), software (equal), validation (equal), visualization (supporting). **Biahong Li:** data curation (equal), formal analysis (supporting), software (equal), supervision (equal). **Wang Xiao:** data curation (equal), formal analysis (supporting), software (equal), validation (supporting). **Chenyan Sui:** investigation (supporting), methodology (equal), resources (equal), software (equal), validation (supporting). **Neng Bao:** investigation (supporting), methodology (equal), software (equal), validation (supporting). **Ruijuan Zhuang:** data curation (equal), formal analysis (equal), validation (equal), writing – original draft (supporting). **Changzheng Gao:** formal analysis (equal), methodology (supporting), resources (equal), software (equal), writing – original draft (supporting). **Xiaoyan Wang:** funding acquisition (equal), investigation (equal), project administration (equal), supervision (equal), writing – review and editing (equal). **Xin Gu:** data curation (equal), formal analysis (equal), funding acquisition (lead), investigation (equal), project administration (lead), supervision (lead), visualization (equal), writing – review and editing (equal).

## Consent

The authors have nothing to report.

## Conflicts of Interest

The authors declare no conflicts of interest.

## Supporting information


Figures S1–S4
Figure S1. p16 was over‐expression in HFD‐induced kidney of ApoE^−/−^ mice.
Figure S2. ABT263 administration inhibited the expression levels of p16 and p53.
Figure S3. ABT263 administration inhibited the fibrosis process in kidney.
Figure S4. p16 knockout remodelled metabolic shift in HFD‐induced kidney.

## Data Availability

All data and materials used in the analysis are available to any researcher for the purposes of reproducing or extending the analysis from corresponding authors.
