# Supplementary figures and images for: p16INK4a Deletion Alleviated Obesity‐Associated Kidney Fibrosis by Regulating Metabolic Reprogramming and the Inflammasome Pathway
Source: J Cell Mol Med. 2025 Mar 13;29(5):e70444. doi: 10.1111/jcmm.70444 (PMC11904428; doi:10.1111/jcmm.70444)

Figure S1

A

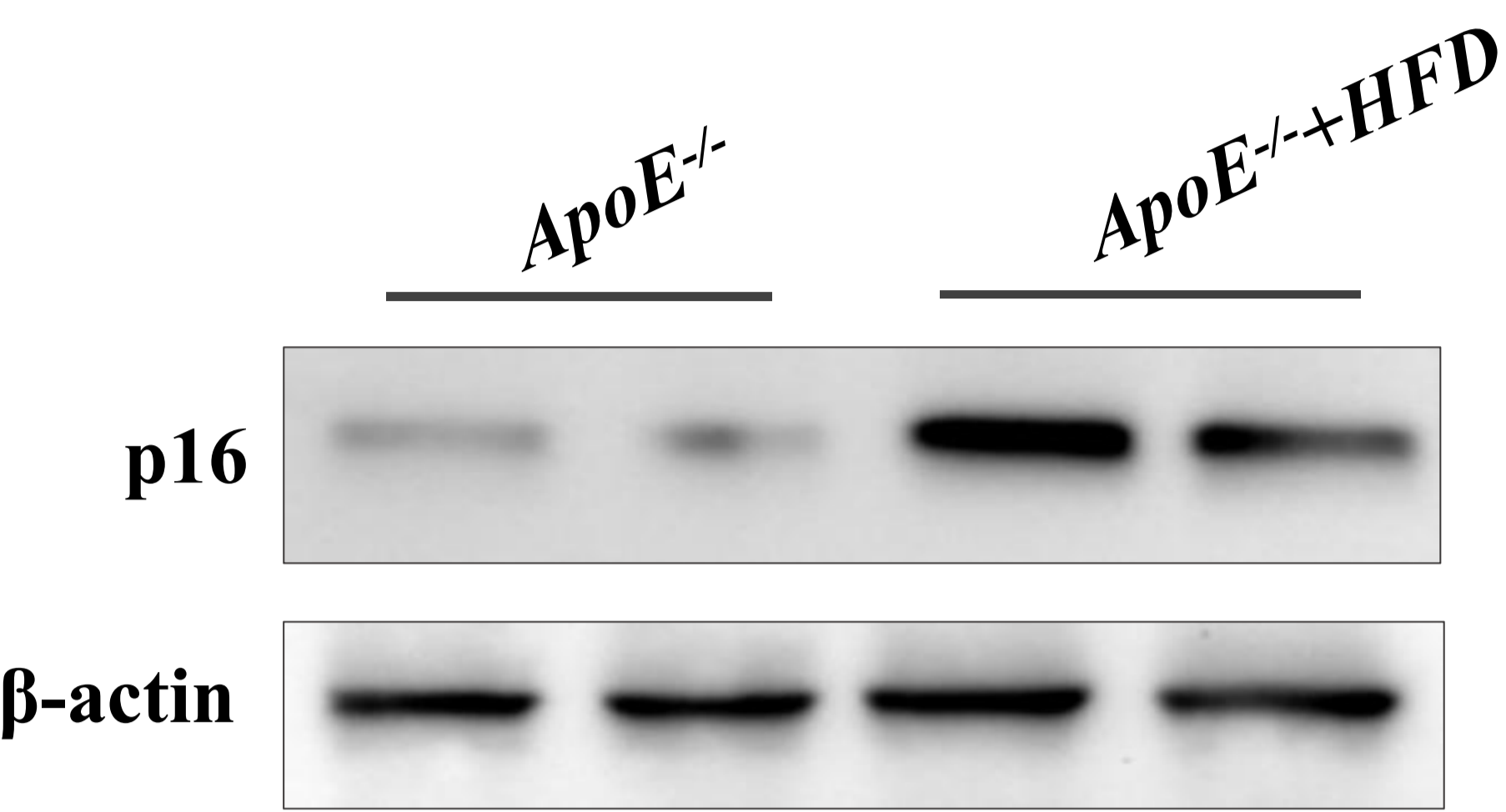

B

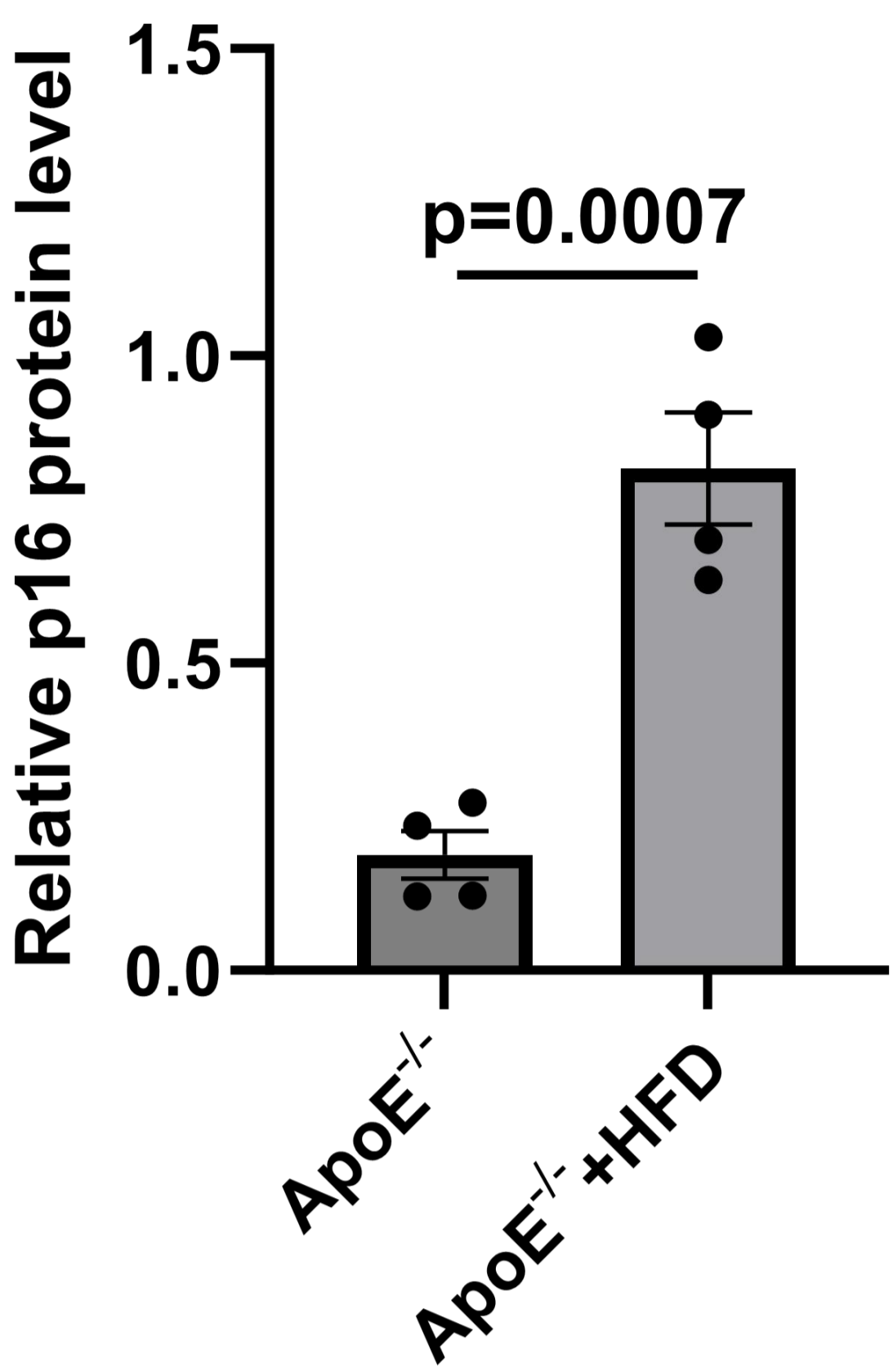

C

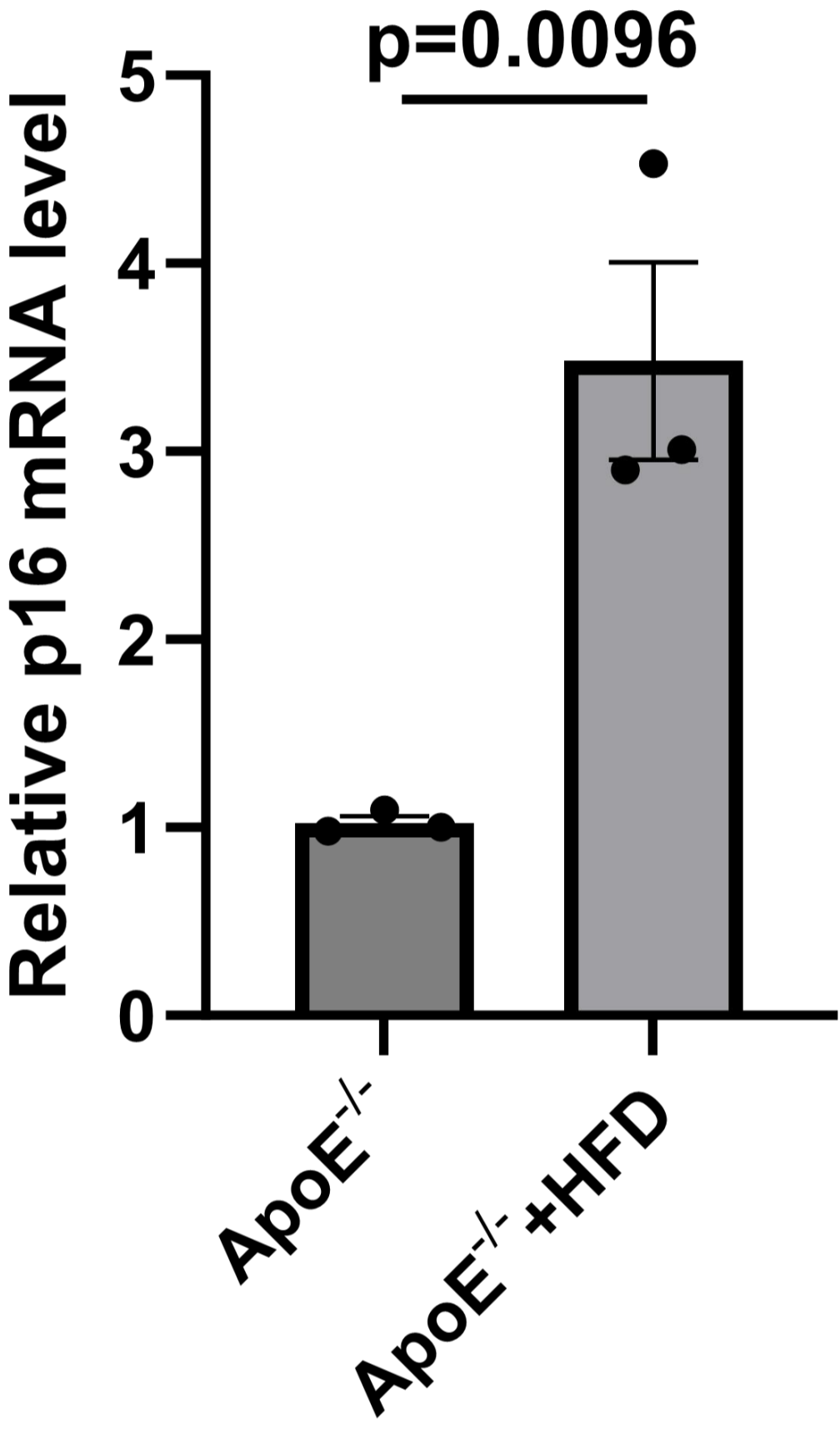

Figure S2

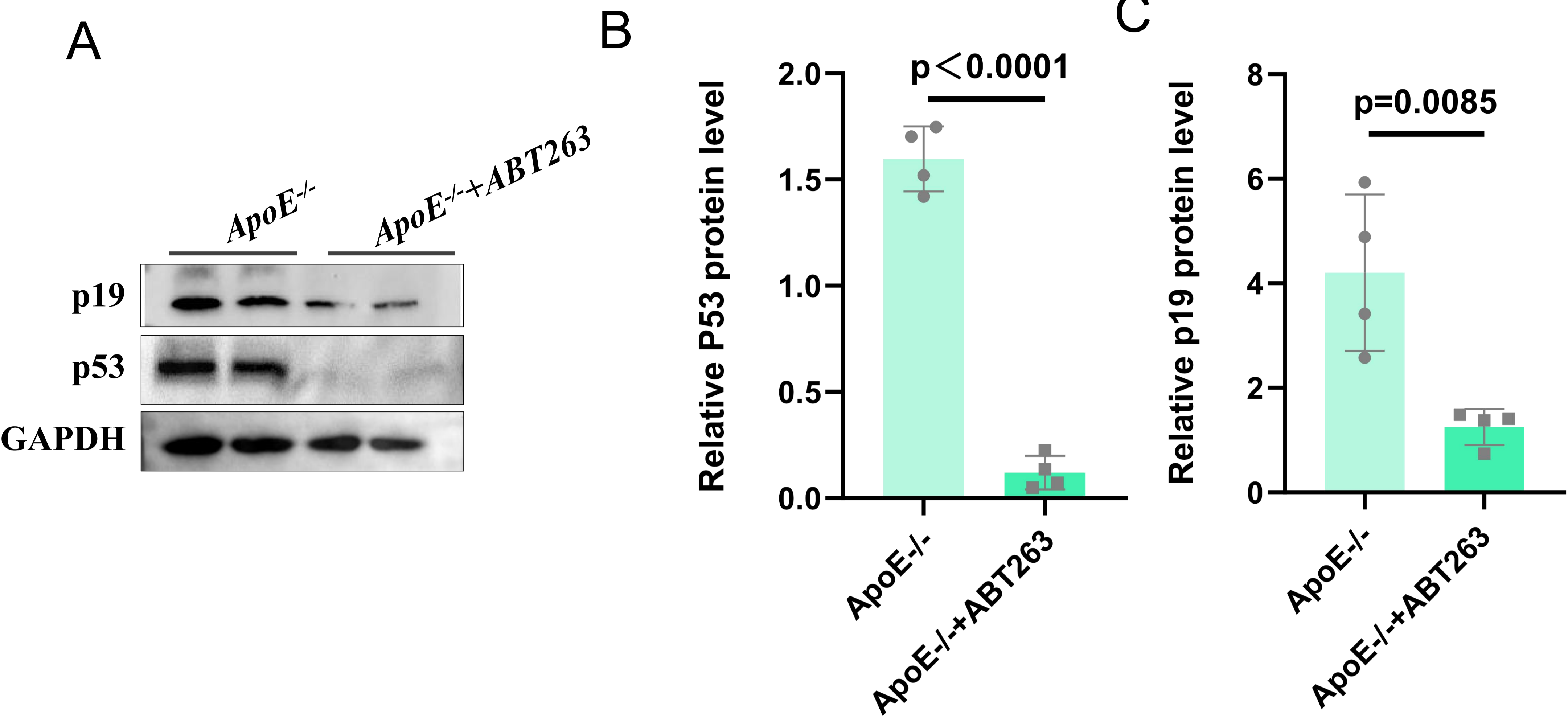

Figure S3

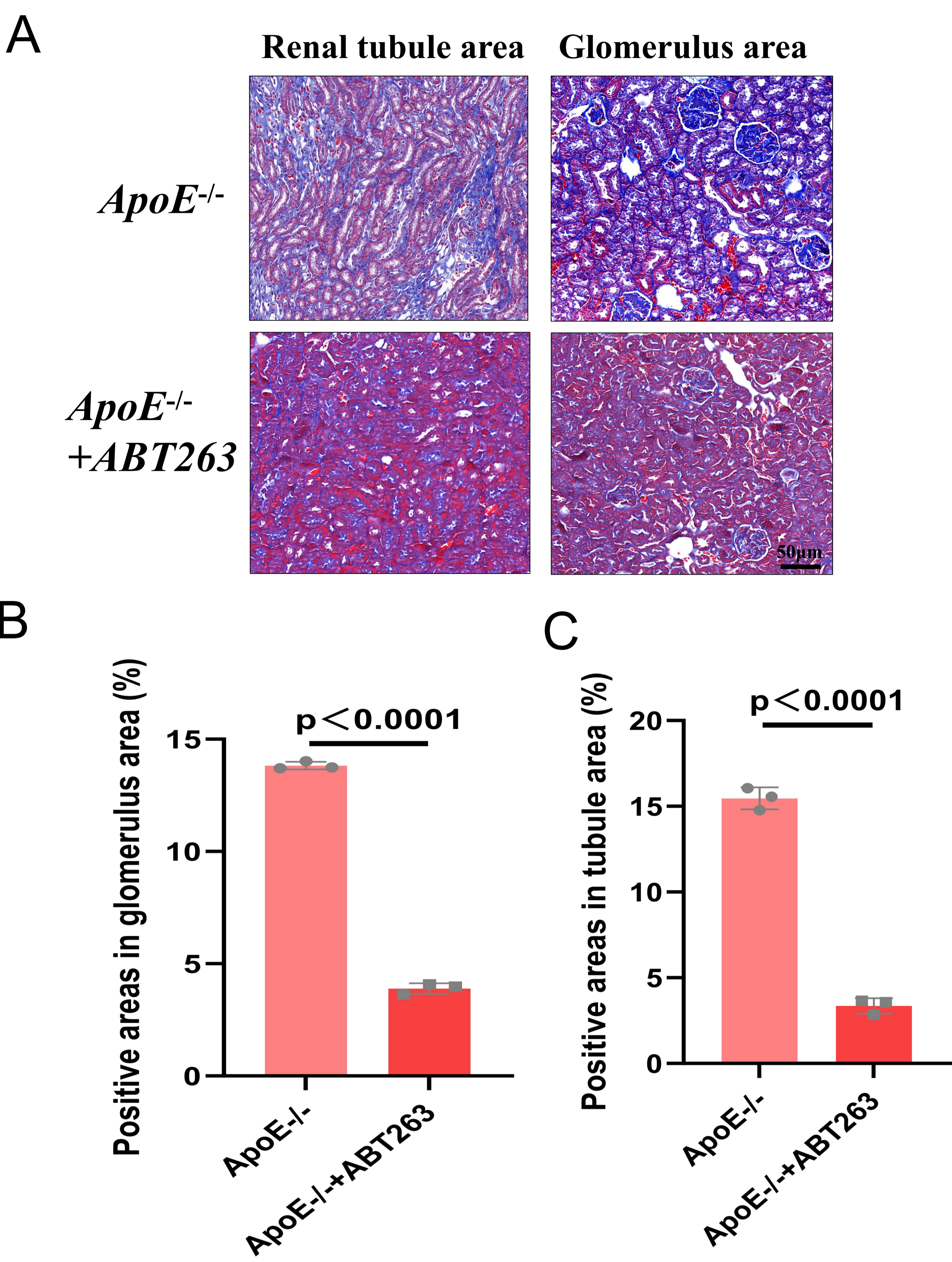

Figure S4

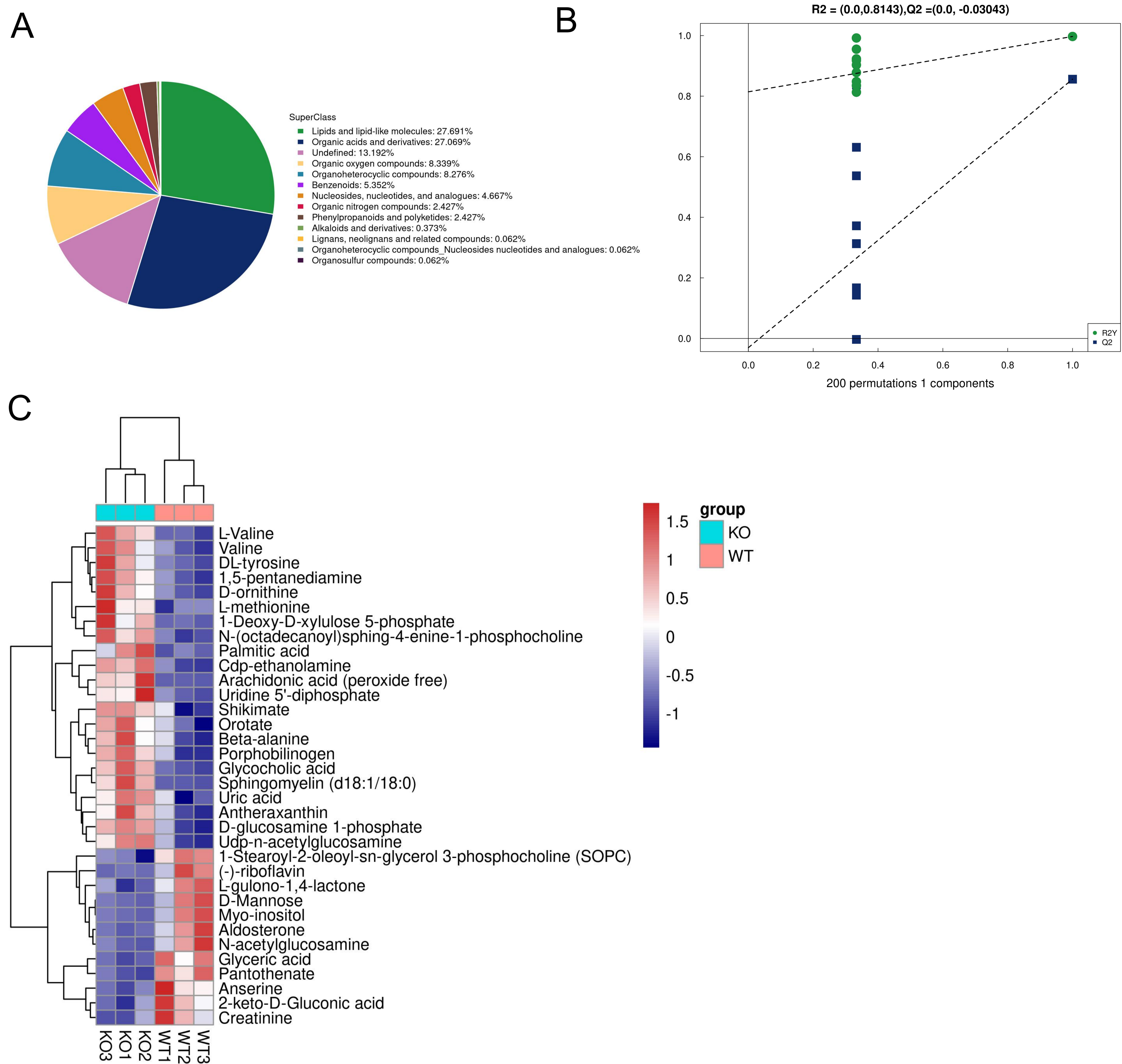

Supplement: Supplementary file 1 — Figures S1–S4 Figure S1. p16 was over‐expression in HFD‐induced kidney of ApoE−/− mice. Figure S2. ABT263 administration inhibited the expression levels of p16 and p53. Figure S3. ABT263 administration inhibited the fibrosis process in kidney. Figure S4. p16 knockout remodelled metabolic shift in HFD‐induced kidney. [file JCMM-29-e70444-s001.pdf]
